# Epalrestat, an Aldose Reductase Inhibitor, Restores Erectile Function in Streptozocin-induced Diabetic Rats

**DOI:** 10.1038/s41443-018-0075-x

**Published:** 2018-09-13

**Authors:** Bai-Bing Yang, Zhi-Wei Hong, Zheng Zhang, Wen Yu, Tao Song, Lei-Lei Zhu, He-Song Jiang, Guo-Tao Chen, Yun Chen, Yu-Tian Dai

**Affiliations:** 1 Department of Andrology, Drum Tower Hospital, Medical School of Nanjing University, Nanjing, 210000 China; 2 Department of Urology, Fujian Provincial Hospital, Fuzhou, 350000 China; 3 Department of Andrology, Jiangsu Provincial Hospital of Traditional Chinese Medicine, Nanjing, 210000 China

**Keywords:** Drug discovery, Metabolic disorders

## Abstract

Epalrestat, an aldose reductase inhibitor (ARI), was adopted to improve the function of peripheral nerves in diabetic patients. The aim of this study was to investigate whether epalrestat could restore the erectile function of diabetic erectile dysfunction using a rat model. From June 2016, 24 rats were given streptozocin (STZ) to induce the diabetic rat model, and epalrestat was administered to ten diabetic erectile dysfunction (DED) rats. Intracavernous pressure (ICP) and mean systemic arterial pressure (MAP), levels of aldose reductase (AR), nerve growth factor (NGF), neuronal nitric oxide synthase (nNOS), α-smooth muscle antigen (α-SMA), and von Willebrand factor (vWF) in the corpus cavernosum were analyzed. We discovered that epalrestat acted on cavernous tissue and partly restored erectile function. NGF and nNOS levels in the corpora were increased after treatment with epalrestat. We also found that the content of α-SMA-positive smooth muscle cells and vWF-positive endothelial cells in the corpora cavernosum were declined. Accordingly, epalrestat might improve erectile function by increasing the upregulation of NGF and nNOS to restore the function of the dorsal nerve of the penis.

## Introduction

Erectile dysfunction (ED) refers to the inability to adequately attain and/or maintain penile erection to allow satisfying sexual intercourse [1], and it is a common complication of diabetes mellitus. The incidence of ED in male patients with diabetes is about three times higher than that in nondiabetic men [2].

In the development of diabetic complications, the increasing of the polyol pathway plays an important role in which sorbitol is accumulated in diabetic neuropathic nerves. Subsequent depletion of myoinositol reduces Na/K-ATPase activity and causes diabetic neuropathy [3]. The degree of sorbitol accumulation has been shown to be significantly linked to the severity of diabetic neuropathy [4]. Epalrestat is one of the inhibitors of aldose reductase (AR), the rate-limiting enzyme of the polyol pathway [5, 6], and was approved for use in the clinic in Japan to improve the functional decrease of peripheral nerves and to increase the number and diameter of nerve fibers [7, 8]. Studies have shown that epalrestat could upregulate the level of nerve growth factor (NGF) in sciatic nerve tissue [9] and improve diabetic wound healing by upregulating NGF in diabetic rats [10]. Interestingly, our previous studies have demonstrated that NGF expression was upregulated in the corpus cavernosum of diabetic erectile dysfunction (DED) rats compared with control rats [11] and that exogenous NGF could partly restore the erectile function of DED rats [12]. Recently, Wu et al. also found that NGF treatment could improve diabetes-induced ED through upregulating the expression of key enzymes in testosterone biosynthesis [13]. In addition, a study indicated that in AR knockout mice, neuronal nitric oxide synthase (nNOS) was upregulated [14], while nNOS is an important enzyme involved in the production of nitric oxide (NO) [15]. Any alteration in the formation of NO from the nerve terminals or the vascular endothelium may influence the corporal smooth muscle relaxation and subsequently reduce the blood flow into corpus cavernosum, finally resulting in erectile dysfunction [16]. Thus, epalrestat showed a potential for treating DED. This study aimed to investigate if epalrestat can improve erectile function in DED rats and to uncover the possible mechanism of this while focusing on the effect on NGF and nNOS in the rat penis.

## Materials and methods

### Animals and treatments

All the experimental protocols were approved by the Institutional Animal Care Committee of Drum Tower Hospital. Thirty 12-week-old male Sprague–Dawley rats (250–300 g) were purchased from Shanghai Slac Laboratory Animal Co. Ltd. (Shanghai, China) in June 2016. All rats were maintained in animal-feeding room with a 12-h light–dark cycle at 25 °C and were provided sufficient food and water. Twenty-four rats were given intraperitoneal injection of freshly prepared STZ (65 mg/kg) for type-I diabetic model. Six rats were given vehicle only (0.1 mol/l citrate/phosphate buffer, pH 4.5) as a control group. Blood glucose was measured 72 h after streptozocin (STZ)/vehicle injection and those with high serum glucose levels (>16.6 mmol/l or 300 mg/dL) were included in this study. Twenty rats with high glucose level were randomly divided into an experimental group (*n* = 10), given intra-gastric epalrestat daily (100 mg/kg/day; Yangtze River Pharmaceutical Group, China) and an untreated DED group (*n* = 10), given intra-gastric normal saline daily at 8:00 am. The level of blood glucose and body weight were measured regularly during the process of study. All rats were maintained for 8 weeks, except for two rats in the DED group and one rat in the epalrestat-treated group died before the end of the study.

### Erectile function assessment and tissue collection

To assess erectile function of rats, the intracarvernous pressure (ICP) and the ratio of the ICP/ mean systemic arterial pressure (MAP) were measured. The evaluation of penile erection with ICP was described by Chen et al. since the year of 1992 [17]. Briefly, under chloral hydrate (0.3 ml/kg) anesthesia, the cavernous nerves were exposed via midline laparotomy. The corpus cavernosum was cannulated with a heparinized (200 U/mL) 25-G needle connected to RM6240B/C multichannel bio-signal collection processing system (Chengdu Implement Company, Chengdu, China). Stimulations were performed at 5 V for approximately 60 s with resting periods of 5 min between subsequent stimulations. The maximum increase of ICP of three stimuli per side was selected for statistical analysis in each animal. ICP was normalized to MAP, which was recorded using a 25-G needle inserted into the aortic bifurcation. After the ICP/MAP assessment, rats were euthanized by an overdose of chloral hydrate and thus the penises were collected. Part of the proximal penis was stored in 4% paraformaldehyde; the remains were stored at −80 °C.

### Western blot

Western blotting was conducted to assess the protein expression of AR, NGF, and nNOS in the penis. The penile tissue that had been stored at −80 °C was powdered and lysed in radioimmunoprecipitation assay buffer (phosphate-buffered solution (PBS), 1% NP-40, 1% Triton X-100, 0.5% sodium deoxycholate, 0.1% sodium dodecyl sulfate, and protease inhibitors). The samples were then homogenized on ice for 10 min and centrifuged at 12,000×g for 15 min at 4 °C. The supernatants were collected and stored at −80 °C. Equal amounts of proteins were electrophoresed on 10% sodium dodecyl sulfate–polyacrylamide gels (SDS-PAGE) and then transferred to a nitrocellulose membrane. The membrane was blocked in Blotto-Tween (10 mmol/L Tris–HCl (pH 8.0), 150 mmol/L NaCl, 5% nonfat dry milk, and 0.05% Tween-20) overnight at 4 °C. The membrane was then incubated with antibodies targeted against AR (Santa Cruz Biotechnology Inc., USA; 1:500), NGF (Santa Cruz Biotechnology Inc., USA; 1:200), and nNOS (Santa Cruz Biotechnology Inc., USA; 1:200) at room temperature for 4 h and then for 1 h at room temperature with goat anti-rabbit (Proteintech Group Inc., USA; 1:2000) secondary antibodies. Detection was performed using enhanced chemiluminescence (ASPEN, Wuhan, China) followed by autoradiography. The densitometric results were quantified using Image-Pro Plus 6.0 software (Media Cybernetics, Inc., Bethesda, MD, USA).

### Immunohistochemistry of ɑ-smooth muscle antigen (ɑSMA), von Willebrand factor (vWF), and nNOS

For immunohistochemistry, the tissue was fixed in 4% paraformaldehyde overnight. Following deparaffinization and rehydration, sections (5 mm) were rinsed for 6 min using PBS. Endogenous peroxidase activity was quenched using 0.3% H_2_O_2_ for 10 min. After 6 min of washing with PBS, the tissue was blocked using 3% bovine serum albumin (BSA) for 30 min and then incubated with anti-nNOS (Santa Cruz Biotechnology Inc., USA; 1:100), anti-ɑ-smooth muscle antigen (Abcam Inc., Hong Kong, China; 1:400), or anti-vWF (Abcam Inc., Hong Kong, China, 1:800) at 4 °C overnight. Sections were then incubated with goat anti-rabbit secondary antibodies (Dako, Glostrup, Denmark; 1:100) for 2 h at room temperature and then counterstained with hematoxylin. Sections incubated without primary antibodies were used as negative controls. Images were captured using a Nikon microscope with a Spot RT color digital camera and digital histomorphometric analysis was performed using Image-Pro Plus 6.0 software.

### Statistical analysis

All data were presented as the mean ± standard deviation. Independent Student’s *t*-test was used to compare the initial and final weight and blood glucose, and one-way ANOVA followed by the LSD test for post hoc comparisons was used to compare the results among three groups. All statistical analyses were carried out with SPSS 24.0 software (SPSS Inc., Chicago, IL, USA). *P* < 0.05 was considered statistically significant.

## Results

### Basic characteristics of rats

The blood glucose levels and body weights are listed in Table 1. After 8 weeks, the body weight of rats in the DED group was decreased (*P* *<* 0.001) and the blood glucose was significantly increased when compared with the controls (*P* *<* 0.001). The body weight and blood glucose of epalrestat-treated rats were not improved after 8-week epalrestat treating (*P* = 0.690 and 0.796, respectively), and both had no difference with those of the diabetic rats (*P* *=* 0.074 and 0.579, respectively).

**Table 1:** Body weight and blood glucose change in control and diabetic rats (mean ± SD)

	Control group	DED group	Epalrestat-treated group	*P* value
Initial weight (g)	271.17 ± 11.25	271.20 ± 15.88	270.10 ± 8.73	0.977
Final weight (g)	425.33 ± 18.52	249.62 ± 19.63*	267.33 ± 19.54*	<0.001
Initial BG (mol/dl)	6.22 ± 0.32	19.51 ± 1.71*	19.52 ± 2.19*	<0.001
Final BG (mol/dl)	6.15 ± 0.52	20.34 ± 1.98*	19.80 ± 2.45*^#^	<0.001

One-way ANOVA followed by the LSD test for post hoc comparisons was used to compare the weight and blood glucose among three groups. Independent Student’s *t*-test was used to compare the initial and final weight and blood glucose of each group

*BG,* blood glucose, *DED,* diabetic erectile dysfunction

*P *<* 0.01 compared with the control group

^#^*P*  <  0.01 compared with the diabetic group

### Effect of epalrestat on erectile function of diabetic rats

**Fig. 1:**
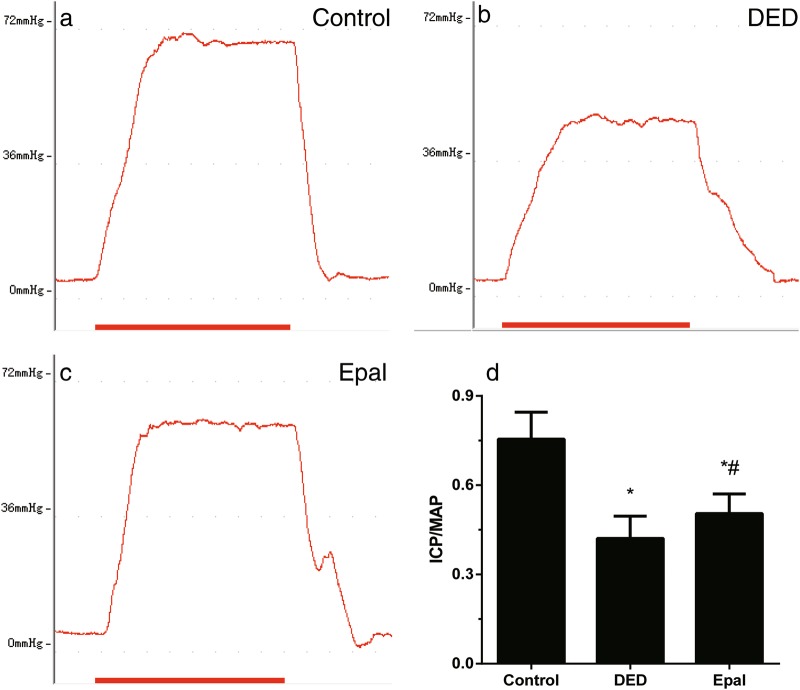
Erectile function evaluation of control rats, diabetic rats, and diabetic rats treated with epalrestat. Intracavernous pressure (ICP) and peak intracavernous pressure/mean system arterial pressure (ICP/MAP). **a** Control group. **b** Diabetic ED group. **c** Epalrestat-treated group. **d** Bar graph of the ICP/MAP ratio. *P *<* 0.01 compared to the control group; ^#^*P* < 0.05 compared to the diabetic group. DED diabetic group, Epal epalrestat-treated group

The ICP/MAP ratio is demonstrated in Fig. 1. When compared with the ratio of normal rats (Fig. 1a), the mean ICP/MAP ratio of untreated diabetic rats (Fig. 1b) had dropped significantly (*P* < 0.001). After the treatment of epalrestat for 8 weeks, the mean ICP/MAP ratio had increased when compared with that of diabetic rats (*P* = 0.036) (Fig. 1c). Epalrestat partially restored the reduction of erectile function but did not bring it back to normal level.

### Protein levels of AR, NGF, and nNOS in the cavernous tissue and dorsal nerve

**Fig. 2:**
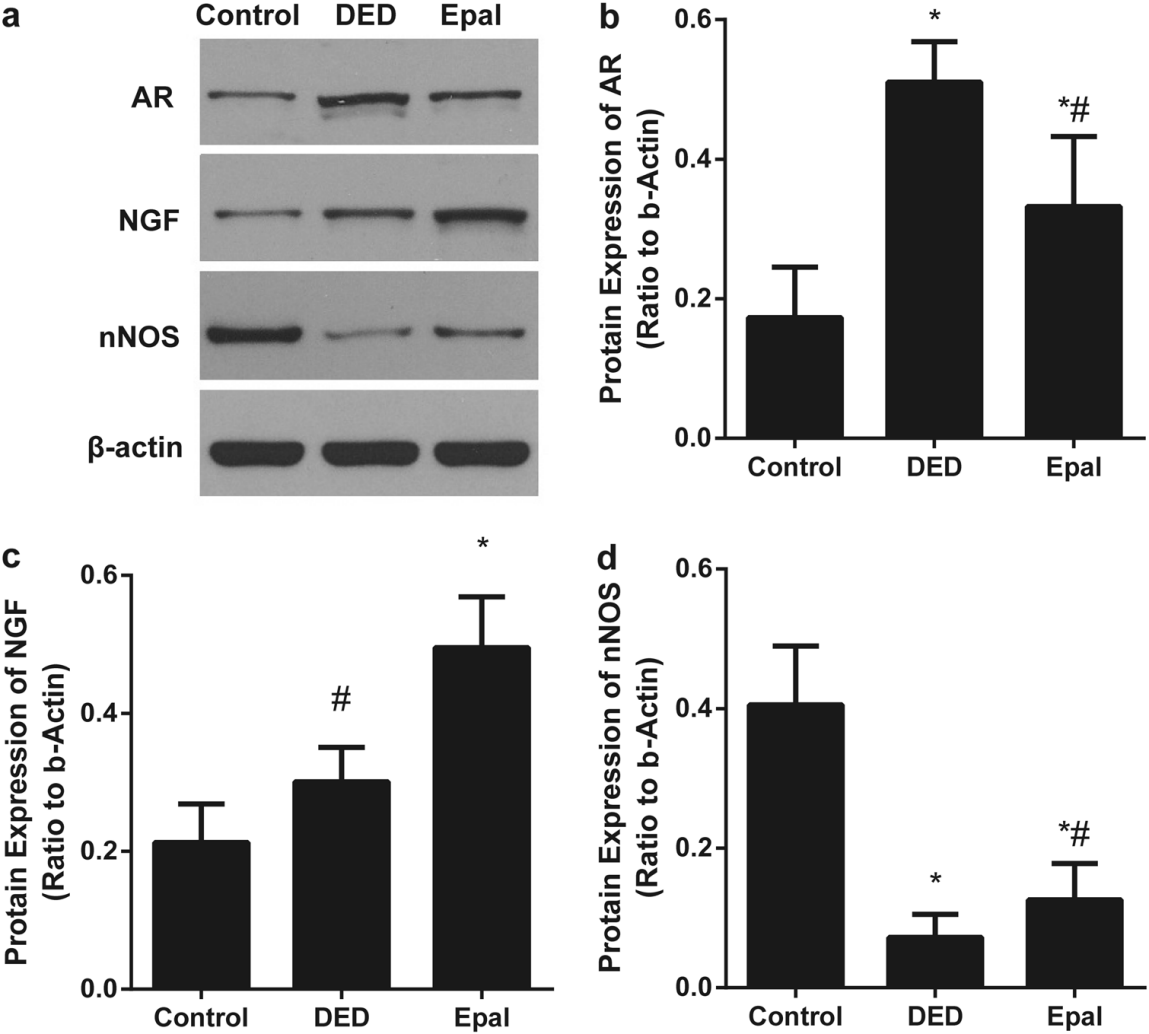
**a** The protein level of AR, NGF, nNOS in the penile tissue. **b** The AR protein level was increased in the diabetic group and decreased in the epalrestat-treated group. **c**, **d** The NGF and nNOS protein levels were decreased in the diabetic controls compared with that in the normal control and increased by epalrestat treatment. *P *<* 0.01 compared to the control group; ^#^*P* *<* 0.05 compared to the diabetic group. DED diabetic group, Epal epalrestat-treated group

Western blot was performed to assess the expression of AR, NGF, and nNOS (Fig. 2). The level of AR protein increased in the untreated diabetic rats when compared with that in normal rats (*P* < 0.001), but the levels of NGF and nNOS were decreased (*P* = 0.001). After 8 weeks of epalrestat treatment, the AR level was decreased (*P* < 0.001), while the NGF and nNOS levels were increased in epalrestat-treated diabetic group compared with that in the untreated diabetic rats (both *P* < 0.001).

### Expression of nNOS in the dorsal nerve

**Fig. 3:**
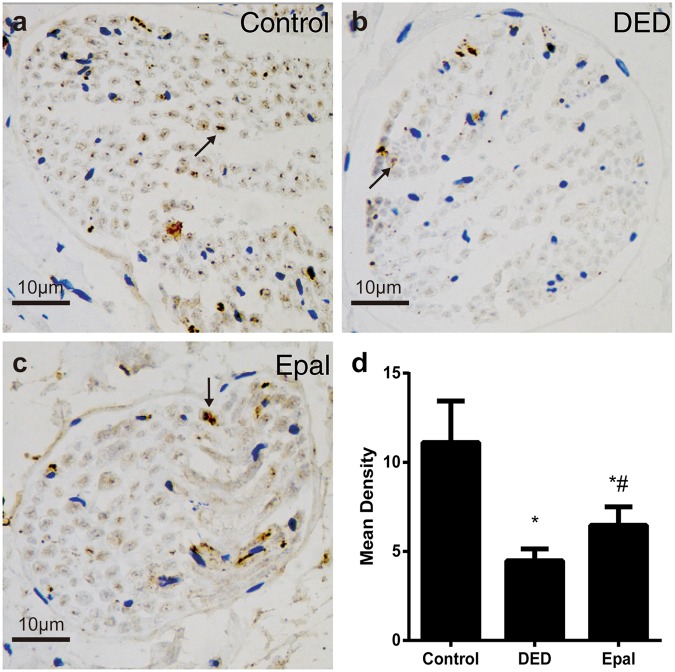
Evaluation of nNOS expression in the nerve fibers of the dorsal nerve. **a** Control group. **b** Diabetic ED group. **c** Epalrestat-treated group. **d** Statistical chart of the nNOS mean density among groups. The arrows indicate the nerve fibers expressing nNOS. *P < 0.01 compared to the control group; ^#^*P* < 0.05 compared to the diabetic group. DED diabetic group, Epal epalrestat-treated group. Scale bars = 10 μm

Immunohistochemical staining was performed to assess nNOS expression in nerve fibers (Fig. 3). The expression of nNOS was significantly greater in the epalrestat-treated group than in the untreated diabetic rats (*P* = 0.012), but still less than the control group (*P* *<* 0.001).

### The effect of epalrestat on the smooth muscle content of the cavernosum

**Fig. 4:**
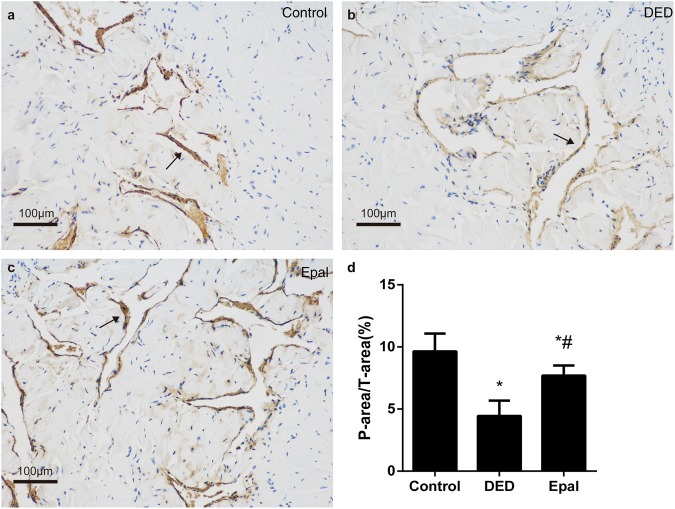
Immunohistochemical staining of α-SMA. **a** Control group. **b** Diabetic ED group. **c** Epalrestat-treated group. **d** Statistical chart of the α-SMA density among groups; the arrows indicate the smooth muscle of cavernosum expressing α-SMA. *P < 0.01 compared to the normal control group; ^#^*P* < 0.05 compared to the diabetic group. DED diabetic group, Epal epalrestat-treated group. α-SMA α-smooth muscle actin. Scale bars = 100 μm

The smooth muscle content (as indicated by α-SMA) was significantly dropped in untreated diabetic rats (Fig. 4b) compared to that in controls (Fig. 4a; *P* < 0.001). After 8 weeks of epalrestat treatment, the smooth muscle content increased when compared to untreated diabetic rats (*P* < 0.001) (Fig. 4c).

### The effect of epalrestat on the endothelium content of the cavernosum

**Fig. 5:**
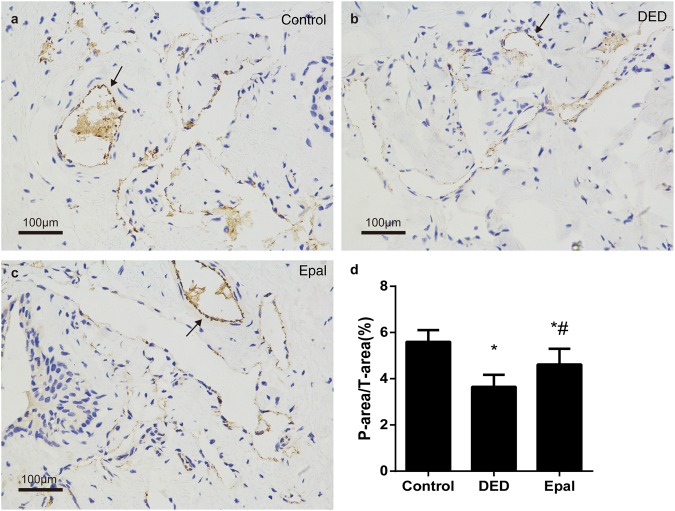
Immunohistochemical staining of vWF. **a** Control group. **b** Diabetic ED group. **c** Epalrestat-treated group. **d** Statistical chart of the vWF density among groups; the arrows indicate the endothelium of cavernosum expressing vWF. *P < 0.01 compared to the normal control group; ^#^*P* < 0.05 compared to the diabetic group. DED diabetic group, Epal epalrestat-treated group, vWF von Willebrand factor. Scale bars = 100 μm

The level of vWF was significantly reduced in the DED group when compared with the control group (*P* < 0.001). After 8 weeks of epalrestat administration, the endothelium content was increased significantly compared to that of the untreated DED group (*P* = 0.005) (Fig. 5).

## Discussion

This study showed that treatment with epalrestat restored ED in STZ-induced diabetic rat. Epalrestat partly restored ICP and increased the ICP/MAP ratio. Two markers, α-SMA and vWF, were used to indicate the content of smooth muscle and endothelium, which played important roles in the erectile function and were usually impaired in DED [18]. α-SMA has been shown to be an excellent molecular marker of smooth muscle cell phenotype [19] and vWF is regarded as a more conventional marker of endothelial cell activation in various diseases affecting the vascular system [20]. The content of α-SMA-positive smooth muscle cells and vWF-positive endothelial cells in the corpora cavernosum was decreased in DED rats, which coincided with previous studies [21, 22], and was partly restored after treatment with epalrestat.

DED is a common diabetic complication and is caused by multiple pathogenic mechanisms, including neural, vascular, endocrine, and metabolic factors [18]. In the diabetic condition, a high concentration of advanced glycosylation end products and free oxygen radicals produced by long periods of high serum glucose lead to the impairment of smooth muscle cells and endothelial cells in the penile cavernosum [23]. Studies have suggested that increased polyol pathway activity resulted in metabolic imbalances which may contribute to the long-term microvascular diabetic complications [24–26]. In hyperglycemic conditions, more than 30% of glucose is metabolized by the polyol pathway, in which increased aldose reductase activity results in the accumulation of sorbitol. Epalrestat is the most frequently used aldose reductase inhibitor for humans at present. Studies have indicated that epalrestat acts in the sciatic nerve, erythrocytes, and ocular tissue in animals and in erythrocytes in humans [9, 27–29]. After treatment with epalrestat, the level of AR in penile cavernosum in the Epal-treated group increased compared with the DED group, suggesting that epalrestat also acted in penis.

NGF, one of the most researched neurotrophins, is an important factor in the process of repair and regeneration of injured nerves [30, 31]. A study has shown that NGF in combination with reconstitutive cavernous nerve guides could enhance erectile responses in rats [32]. In this study, the level of NGF in rats treated with epalrestat was further increased compared to that of the DED group, indicating that epalrestat could induce the expression of more NGF in the penile dorsal nerve of the diabetic rat, which was in accordance with Ohi’s [9] and Nakagaki’s [10] results. The possible mechanism underlying the upregulating effect of epalrestat on NGF may be the prevention of the overconsumption of NADPH in the polyol pathway. In hyperglycemic conditions, NADPH is overused through accelerated flux in the polyol pathway and secondarily decreases the NADPH ratio in the pentose phosphate pathway, which subsequently decreases GSH, consequently leading to the reduction of NGF expression [33].

NOS plays an important role in maintaining erectile function [15]. NO released within the corpora cavernosa leads to the relaxation of the smooth muscle, initiating the hemodynamic changes of penile cavernosum, and contributing to the maintenance of tumescence [34]. The specific NOS inhibitor L-NAME completely blocked the salutary effects of a potent ARI on nerve conduction velocities in STZ-rats, without affecting the accumulation of sorbitol [3]. In this study, the expression of nNOS in the dorsal nerve of the penis was higher after treatment with epalrestat than in DED rats, suggesting that epalrestat increased the level of nNOS and facilitated the regeneration of the nNOS-containing nerve fibers in the rat penile dorsal nerve, which has never been reported before.

We also found that epalrestat increased the smooth muscle content and the endothelium content in the cavernosum. Gu et al. [35] demonstrated that epalrestat could reverse vascular remodeling, perhaps through the inhibition of the Ang II-induced signal pathway or AR-involved oxidative stress. Epalrestat has also been reported to show a therapeutic effect on macroangiopathy through inhibiting PKC activity and oxidative stress. Thus, the effect of epalrestat on vascular mechanisms and oxidative stress needs to be explored further.

Some limitations exist in this study. The diabetic rats were induced with STZ, representing type-I diabetes mellitus. As we focused on the neural effect of epalrestat, further studies on the vascular effect and the effects on oxidative stress under different glucose levels are recommended. As this study was carried out in vivo, further study on cells in vitro may offer more evidence on the therapeutic effect of epalrestat. Although epalrestat was generally safe in clinical trials [36–38], the effective dose on erectile function and the way to combinate with other drugs treating ED is still to be explored, both in rodents and in humans.

## Conclusions

Epalrestat is an AR inhibitor used in humans to improve the declined function of peripheral nerves and increase the number and diameter of nerve fibers of diabetic patients. It shows promise in the treatment of DED, perhaps through the upregulation of NGF and the recovery of nNOS expression in the dorsal nerve. Though only tested in animal experiments and with the vascular and oxidative stress effects still to be verified, our results suggested a new kind of drug to treat DED. Further studies are recommended for the restoration of erectile function in DED patients.

## References

[1] Lue TF, Giuliano F, Montorsi F, Rosen RC, Andersson KE (2004). Summary of the recommendations on sexual dysfunctions in men. J Sex Med.

[2] Richardson D, Vinik A (2002). Etiology and treatment of erectile failure in diabetes mellitus. Curr Diab Rep.

[3] Stevens MJ, Dananberg J, Feldman EL, Lattimer SA, Kamijo M (1994). The linked roles of nitric oxide, aldose reductase and, (Na + ,K + )-ATPase in the slowing of nerve conduction in the streptozotocin diabetic rat. J Clin Invest.

[4] Kikkawa R, Hatanaka I, Yasuda H, Kobayashi N, Shigeta Y (1983). Effect of a new aldose reductase inhibitor, (E)-3-carboxymethyl-5-[(2E)-methyl-3-phenylpropenylidene]rhodanine (ONO-2235) on peripheral nerve disorders in streptozotocin-diabetic rats. Diabetologia.

[5] Chalk C, Benstead TJ, Moore F. Aldose reductase inhibitors for the treatment of diabetic polyneuropathy. *Cochrane Datab Syst Rev* 2007;(4):CD004572. 10.1002/14651858.CD004572.pub2 PMC8406996 17943821

[6] Ramirez MA, Borja NL (2008). Epalrestat: an aldose reductase inhibitor for the treatment of diabetic neuropathy. Pharmacotherapy.

[7] Baba M, Kimura K, Suda T, Yagihashi S, Aomori Diabetic Study G. (2006). Three-year inhibition of aldose reductase on development of symptomatic neuropathy in diabetic patients. J Peripher Nerv Syst.

[8] Sasaki H, Naka K, Kishi Y, Furuta M, Sanke T (1997). The absence of synergism between the effects of an aldose reductase inhibitor, epalrestat, and a vasodilator, cilostazol, on the nerve conduction slowing and the myelinated fiber atrophy in streptozotocin-induced diabetic rats. Exp Neurol.

[9] Ohi T, Saita K, Furukawa S, Ohta M, Hayashi K (1998). Therapeutic effects of aldose reductase inhibitor on experimental diabetic neuropathy through synthesis/secretion of nerve growth factor. Exp Neurol.

[10] Nakagaki O, Miyoshi H, Sawada T, Atsumi T, Kondo T (2013). Epalrestat improves diabetic wound healing via increased expression of nerve growth factor. Exp Clin Endocrinol Diabetes.

[11] Chen Y, Yang R, Yao L, Sun Z, Wang R (2007). Differential expression of neurotrophins in penises of streptozotocin-induced diabetic rats. J Androl.

[12] Dai YT, Chen Y, Yao LS, Yang R, Sun ZY (2005). Expression of nerve growth factor in cavernous tissue and its effects on the treatment of rats with diabetic erectile dysfunction. Zhonghua Nan Ke Xue.

[13] Wu Y, Yang C, Meng F, Que F, Xiao W, Althof S, et al. Nerve growth factor improves the outcome of type 2 diabetes-induced hypotestosteronemia and erectile dysfunction. *Reprod Sci*. 2018, [Epub ahead of print]. 10.1177/1933719118773421. 10.1177/1933719118773421 29724155

[14] Yeung PKK, Lai AKW, Son HJ, Zhang X, Hwang O (2017). Aldose reductase deficiency leads to oxidative stress-induced dopaminergic neuronal loss and autophagic abnormality in an animal model of Parkinson’s disease. Neurobiol Aging.

[15] Musicki B, Ross AE, Champion HC, Burnett AL, Bivalacqua TJ (2009). Posttranslational modification of constitutive nitric oxide synthase in the penis. J Androl.

[16] Bivalacqua TJ, Usta MF, Champion HC, Kadowitz PJ, Hellstrom WJ (2003). Endothelial dysfunction in erectile dysfunction: role of the endothelium in erectile physiology and disease. J Androl.

[17] Chen KK, Chan JY, Chang LS, Chen MT, Chan SH (1992). Intracavernous pressure as an experimental index in a rat model for the evaluation of penile erection. J Urol.

[18] Moore CR, Wang R (2006). Pathophysiology and treatment of diabetic erectile dysfunction. Asian J Androl.

[19] McHugh KM (1995). Molecular analysis of smooth muscle development in the mouse. Dev Dyn.

[20] Lim HS, Lip GY, Blann AD (2004). Plasma von Willebrand factor and the development of the metabolic syndrome in patients with hypertension. J Clin Endocrinol Metab.

[21] De Young LX, Domes T, Lim K, Carson J, Brock GB (2008). Endothelial rehabilitation: the impact of chronic PDE5 inhibitors on erectile function and protein alterations in cavernous tissue of diabetic rats. Eur Urol.

[22] Garcia MM, Fandel TM, Lin G, Shindel AW, Banie L (2010). Treatment of erectile dysfunction in the obese type 2 diabetic ZDF rat with adipose tissue-derived stem cells. J Sex Med.

[23] Vlassara H, Fuh H, Makita Z, Krungkrai S, Cerami A (1992). Exogenous advanced glycosylation end products induce complex vascular dysfunction in normal animals: a model for diabetic and aging complications. Proc Natl Acad Sci USA.

[24] Yabe-Nishimura C (1998). Aldose reductase in glucose toxicity: a potential target for the prevention of diabetic complications. Pharmacol Rev.

[25] Okayama N, Omi H, Okouchi M, Imaeda K, Kato T (2002). Mechanisms of inhibitory activity of the aldose reductase inhibitor, epalrestat, on high glucose-mediated endothelial injury: neutrophil-endothelial cell adhesion and surface expression of endothelial adhesion molecules. J Diabetes Complicat.

[26] Hamada Y, Nakamura J (2004). Clinical potential of aldose reductase inhibitors in diabetic neuropathy. Treat Endocrinol.

[27] Steele JW, Faulds D, Goa KL (1993). Epalrestat. A review of its pharmacology, and therapeutic potential in late-onset complications of diabetes mellitus. Drugs Aging.

[28] Terashima H, Hama K, Yamamoto R, Tsuboshima M, Kikkawa R (1984). Effects of a new aldose reductase inhibitor on various tissues in vitro. J Pharmacol Exp Ther.

[29] Hayashi R, Hayakawa N, Makino M, Nagata M, Kakizawa H (1998). Changes in erythrocyte sorbitol concentrations measured using an improved assay system in patients with diabetic complications and treated with aldose reductase inhibitor. Diabetes Care.

[30] Huang EJ, Reichardt LF (2001). Neurotrophins: roles in neuronal development and function. Annu Rev Neurosci.

[31] Calcutt NA, Freshwater JD, Hauptmann N, Taylor EM, Mizisin AP (2006). Protection of sensory function in diabetic rats by Neotrofin. Eur J Pharmacol.

[32] Burnett AL, Lue TF (2006). Neuromodulatory therapy to improve erectile function recovery outcomes after pelvic surgery. J Urol.

[33] Suzuki T, Sekido H, Kato N, Nakayama Y, Yabe-Nishimura C (2004). Neurotrophin-3-induced production of nerve growth factor is suppressed in Schwann cells exposed to high glucose: involvement of the polyol pathway. J Neurochem.

[34] Bella AJ, Lin G, Lin CS, Hickling DR, Morash C (2009). Nerve growth factor modulation of the cavernous nerve response to injury. J Sex Med.

[35] Gu J, Wang JJ, Yan J, Cui CF, Wu WH (2011). Effects of lignans extracted from *Eucommia ulmoides* and aldose reductase inhibitor epalrestat on hypertensive vascular remodeling. J Ethnopharmacol.

[36] Hotta N, Sakamoto N, Shigeta Y, Kikkawa R, Goto Y (1996). Clinical investigation of epalrestat, an aldose reductase inhibitor, on diabetic neuropathy in Japan: multicenter study. Diabetic Neuropathy Study Group in Japan. J Diabetes Complicat.

[37] Hotta N, Akanuma Y, Kawamori R, Matsuoka K, Oka Y (2006). Long-term clinical effects of epalrestat, an aldose reductase inhibitor, on diabetic peripheral neuropathy: the 3-year, multicenter, comparative aldose reductase inhibitor-diabetes complications trial. Diabetes Care.

[38] Hotta N, Kawamori R, Fukuda M, Shigeta Y, Aldose Reductase Inhibitor-Diabetes Complications Trial Study G. (2012). Long-term clinical effects of epalrestat, an aldose reductase inhibitor, on progression of diabetic neuropathy and other microvascular complications: multivariate epidemiological analysis based on patient background factors and severity of diabetic neuropathy. Diabet Med.

