# Correction: Epalrestat, an Aldose Reductase Inhibitor, Restores Erectile Function in Streptozocin-induced Diabetic Rats

**DOI:** 10.1038/s41443-018-0106-7

**Published:** 2019-02-04

**Authors:** Bai-Bing Yang, Zhi-Wei Hong, Zheng Zhang, Wen Yu, Tao Song, Lei-Lei Zhu, He-Song Jiang, Guo-Tao Chen, Yun Chen, Yu-Tian Dai

**Affiliations:** 1 Department of Andrology, Drum Tower Hospital, Medical School of Nanjing University, Nanjing, 210000 China; 2 Department of Urology, Fujian Provincial Hospital, Fuzhou, 350000 China; 3 Department of Andrology, Jiangsu Provincial Hospital of Traditional Chinese Medicine, Nanjing, 210000 China

**Keywords:** Drug discovery, Metabolic disorders

**Correction to: International Journal of Impotence Research;**

10.1038/s41443-018-0075-x;

published online 13 September 2018

This Article was originally published under Nature Research’s License to Publish, but has now been made available under a CC BY 4.0 license. The PDF and HTML versions of the Article have been modified accordingly.

